# Jacaric acid inhibits the growth of murine macrophage-like leukemia PU5-1.8 cells by inducing cell cycle arrest and apoptosis

**DOI:** 10.1186/s12935-015-0246-5

**Published:** 2015-09-29

**Authors:** Wai Nam Liu, Kwok Nam Leung

**Affiliations:** Biochemistry Programme, School of Life Sciences, The Chinese University of Hong Kong, Shatin, HKSAR, China

**Keywords:** Apoptosis, Cell cycle arrest, Conjugated linolenic acids, Jacaric acid, Macrophage-like leukemia, PU5-1.8 cells

## Abstract

**Background:**

Conjugated linolenic acids (CLN) refer to the positional and geometric isomers of octadecatrienoic acids with three conjugated double bonds (C18:3). Previous researches have demonstrated that CLN can inhibit the growth of a wide variety of cancer cells, whereas the modulatory effect of CLN on various myeloid leukemia cells remains unclear. This study aims at demonstrating the in vitro anti-tumor effect and action mechanisms of jacaric acid, a CLN isomer which is present in jacaranda seed oil, on the murine macrophage-like leukemia PU5-1.8 cells.

**Methods and results:**

It was found that jacaric acid inhibited the proliferation of PU5-1.8 cells in a time- and concentration-dependent manner, as determined by the MTT reduction assay and by using CyQUANT^®^ NF Cell Proliferation Assay Kit, while it exerted minimal cytotoxicity on normal murine cells. Besides, the reactive oxygen species production in jacaric acid-treated PU5-1.8 cells was elevated in a concentration-dependent mannar. Flow cytometric analysis revealed the induction of G_0_/G_1_ cell cycle arrest, accompanied by a decrease in CDK2 and cyclin E proteins. Jacaric acid also triggered apoptosis as reflected by induction of DNA fragmentation, phosphatidylserine externalization, mitochondrial membrane depolarization, up-regulation of pro-apoptotic Bax protein and down-regulation of anti-apoptotic Bcl-2 and Bcl-x_L_ proteins.

**Conclusions:**

Our results demonstrated the growth-inhibitory effect of jacaric acid on PU5-1.8 cells through inducing cell cycle arrest and apoptosis, while exhibiting minimal cytotoxicity to normal murine cells. Therefore, jacaric acid is a potential candidate for the treatment of some forms of myeloid leukemia with minimal toxicity and fewer side effects.

## Background

Leukemia is a cancer of the blood or bone marrow due to the uncoupling or imbalance of the proliferation and differentiation of hematopoietic stem cells (HSC) [1]. As a result, the immature precursor cells accumulate and retain their proliferative ability without completing the differentiation program [2]. Conventional modalities for leukemia treatment include chemotherapy, radiotherapy or HSC transplantation, and these are known to be accompanied with many adverse side effects [3–5]. Therefore, there is an immense interest in the searching of novel therapeutic compounds with high efficacy and minimal toxicity, especially those derived from natural or dietary sources [6].

Conjugated fatty acids (CFA) refer to different positional and geometric isomers of polyunsaturated fatty acids (PUFA) that contain conjugated double bonds, in which two carbon–carbon double bonds in the fatty acid acyl chain are separated by one carbon–carbon single bond [7]. The most common naturally-occurring CFA include conjugated linoleic acids (CLA) from ruminant meats and dairy products [8], and conjugated linolenic acids (CLN) from plant seed oils [9]. CFA have attracted considerable attention in recent years because of their unique properties, relatively high potency, diverse metabolic effects and potentially beneficial effects on human health [10, 11]. Among all CFA, isomers of linoleic acid with two conjugated double bonds (CLA) have been most extensively studied in relation to their metabolism and physiological effects [10]. Nevertheless, CLA occur in natural food in less than 1 % of total lipids [9, 12]. In contrast, isomers of linolenic acid with three conjugated double bonds (CLN) are present in some plant seed oils at much higher concentrations (30–70 %) [9, 12]. CLN are a mixture of positional and geometric isomers of octadecatrienoic acid (C18:3). Three 8, 10, 12-triene isomers and four 9, 11, 13-triene isomers have been reported, with various *cis*- or *trans*- isomeric combinations are found in a number of plant seed oils, including the seed oils of pomegranate, bitter gourd, catalpa, pot marigold and jacaranda [13]. Recent researches have shown that CLN isomers possess diverse physiological and pharmacological activities, such as modulation of fat storage, promotion of the proliferation of normal keratinocytes, antioxidative, chemopreventive and anti-tumor properties etc. [14, 15]. Accumulating evidences have demonstrated the growth-inhibitory effects of CLN on a wide variety of human cancer cell lines in vitro, which include hepatoma HepG2 cells, lung adenocarcinoma A549 cells, breast adenocarcinoma MCF-7 cells, stomach tubular adenocarcinoma MKN-7 cells, colon carcinoma DLD-1 cells, bladder cancer T24 cells and prostate cancer LNCaP and PC-3 cells [9, 12, 16, 17]. Nevertheless, their anti-tumor effect, action mechanisms and therapeutic potential on various types of myeloid leukemia cells remain poorly understood.

**Fig. 1:**
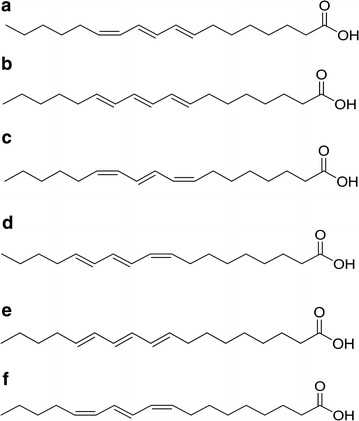
Chemical structures of conjugated linolenic acid (CLN) isomers used in this study. **a** α-calendic acid (8*E*, 10*E*, 12*Z*-octadecatrienoic acid), **b** β-calendic acid (8*E*, 10*E*, 12*E*-octadecatrienoic acid), **c** jacaric acid (8*Z*, 10*E*, 12*Z*-octadecatrienoic acid), **d** α-eleostearic acid (9*Z*, 11*E*, 13*E*-octadecatrienoic acid), **e** β-eleostearic acid (9*E*, 11*E*, 13*E*-octadecatrienoic acid) and **f** punicic acid (9*Z*, 11*E*, 13*Z*-octadecatrienoic acid)

In the present study, we compared the anti-proliferative effect of different CLN isomers on the murine macrophage-like leukemia PU5-1.8 cells. It was found that jacaric acid (8*Z*, 10*E*, 12*Z*-octadecatrienoic acid, Fig. 1) is the most potent CLN isomer that can significantly suppress the in vitro growth of PU5-1.8 cells. Our results show that the anti-leukemic actions of jacaric acid on PU5-1.8 cells might due to the triggering of cell cycle arrest and by inducing apoptosis of the leukemia cells.

## Results

### Conjugated linolenic acids inhibit the proliferation of the murine macrophage-like leukemia cells

**Fig. 2:**
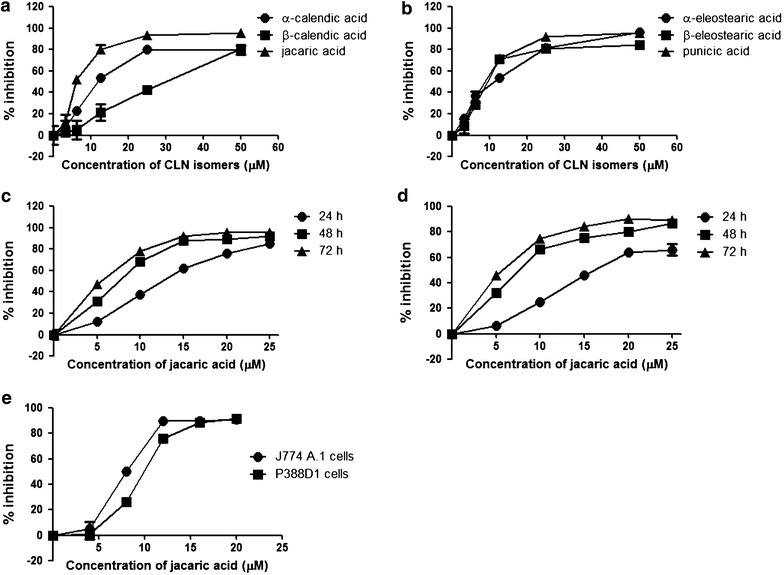
Anti-proliferative effect of CLN isomers on murine macrophage-like leukemia cells. **a**, **b** The murine macrophage-like PU5-1.8 cells were incubated with the solvent control (0.01 % ethanol) or different concentrations of CLN isomers at 37 °C for 48 h. Cell growth was determined by the MTT reduction assay and the results were expressed as mean % inhibition of cell proliferation ± SE. **c**, **d** PU5-1.8 cells were incubated with the solvent control (0.01 % ethanol) or different concentrations of jacaric acid at 37 °C for various periods of time. Cell proliferation was determined by the MTT reduction assay (**c**) or CyQUANT^®^ NF Cell Proliferation Assay Kit (**d**) and the results were expressed as mean % inhibition of cell proliferation ± SE. **e** The murine macrophage-like J774 A.1 and P388D1 cells were incubated with the solvent control (0.01 % ethanol) or different concentrations of jacaric acid at 37 °C for 48 h. Cell growth was determined by the MTT reduction assay, and the results were expressed as mean % inhibition of cell proliferation ± SE

**Fig. 3:**
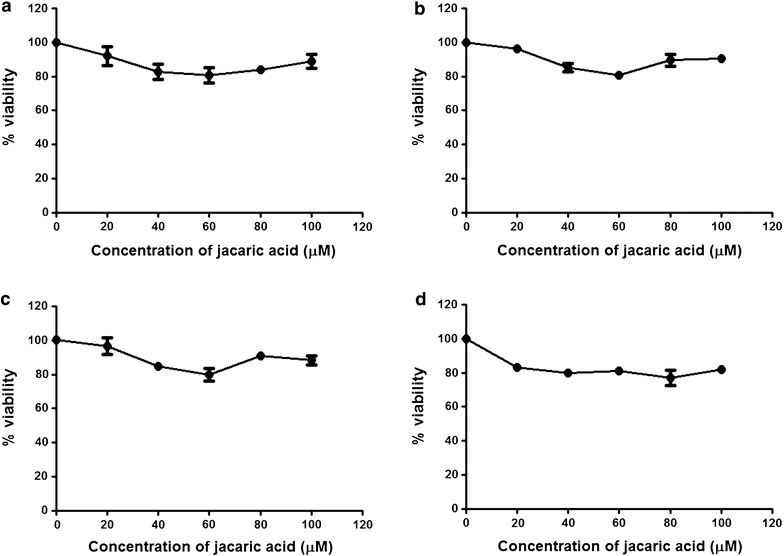
Effect of jacaric acid on the viability of normal murine cells. Murine bone marrow cells (**a**), peritoneal macrophages (**b**), splenocytes (**c**) and thymocytes (**d**) were incubated with the solvent control (0.1 % ethanol) or different concentrations of jacaric acid at 37 °C for 48 h. Cell viability was determined by the MTT reduction assay and the results were expressed as mean % viability ± SE

To measure the anti-proliferative effect of CLN isomers on PU5-1.8 cells, the MTT reduction assay was performed. As shown in Fig. 2a, b, all the six CLN isomers were found to exhibit an inhibitory effect on the proliferation of PU5-1.8 cells in a concentration-dependent manner, with an estimated 50 % inhibitory concentration (IC_50_) ranging from 6 μM (for jacaric acid) to 29.69 μM (for β-calendic acid) after 48 h treatment (Table 1). Interestingly, the solvent control (up to 0.1 % v/v ethanol) did not exert any significant inhibitory effect on PU5-1.8 cells (<5 % inhibition, data not shown). Since jacaric acid was found to be the most potent isomer among all the six CLN isomers tested, it was chosen as the specific target for further investigation of its anti-leukemic activity on PU5-1.8 cells. Figure 2c shows that jacaric acid inhibited the growth of PU5-1.8 cells in a time- and concentration-dependent manner as measured by the MTT assay, and the growth-inhibitory effect of jacaric acid on PU5-1.8 cells was confirmed using the CyQUANT^®^ NF Cell Proliferation Assay Kit, which specifically measures cell proliferation (Fig. 2d). To determine whether the anti-proliferative effect of jacaric acid is cell line specific or not, two other murine macrophage-like leukemia cell lines, including J774 A.1 cells and P388D1 cells, were also examined. It was found that jacaric acid could also exhibit an anti-proliferative effect on J774 A.1 cells and P388D1 cells in a concentration-dependent manner as determined by the MTT assay (Fig. 2e), suggesting that the anti-proliferative effect of jacaric acid is not tumor cell line specific. Intriguingly, jacaric acid exhibited little, if any, direct cytotoxicity on normal primary myeloid cells such as murine bone marrow cells, peritoneal macrophages, splenocytes and thymocytes, as the percentage of cell viability of the cells remained >80 % even when the cells were incubated with 100 µM jacaric acid for 48 h (Fig. 3).

**Table 1:** The estimated 50 % inhibitory concentration (IC_50_) of different CLN isomers for PU5-1.8 cells

CLN isomer	IC_50_ (µM) at 48 h
α-Calendic acid (8*E*, 10*E*, 12*Z*-octadecatrienoic acid)	12.11 ± 1.35
β-Calendic acid (8*E*, 10*E*, 12*E*-octadecatrienoic acid)	29.69 ± 4.04
Jacaric acid (8*Z*, 10*E*, 12*Z*-octadecatrienoic acid)	6.00 ± 0.87
α-Eleostearic acid (9*Z*, 11*E*, 13*E*-octadecatrienoic acid)	10.74 ± 1.33
β-Eleostearic acid (9*E*, 11*E*, 13*E*-octadecatrienoic acid)	9.57 ± 0.75
Punicic acid (9*Z*, 11*E*, 13*Z*-octadecatrienoic acid)	8.40 ± 1.48

The IC_50_ value is the concentration of CLN isomer that inhibits the proliferation of the murine leukemia PU5-1.8 cells by 50 % after 48-h treatment

### Jacaric acid enhances the generation of reactive oxygen species (ROS) in PU5-1.8 cells

**Fig. 4:**
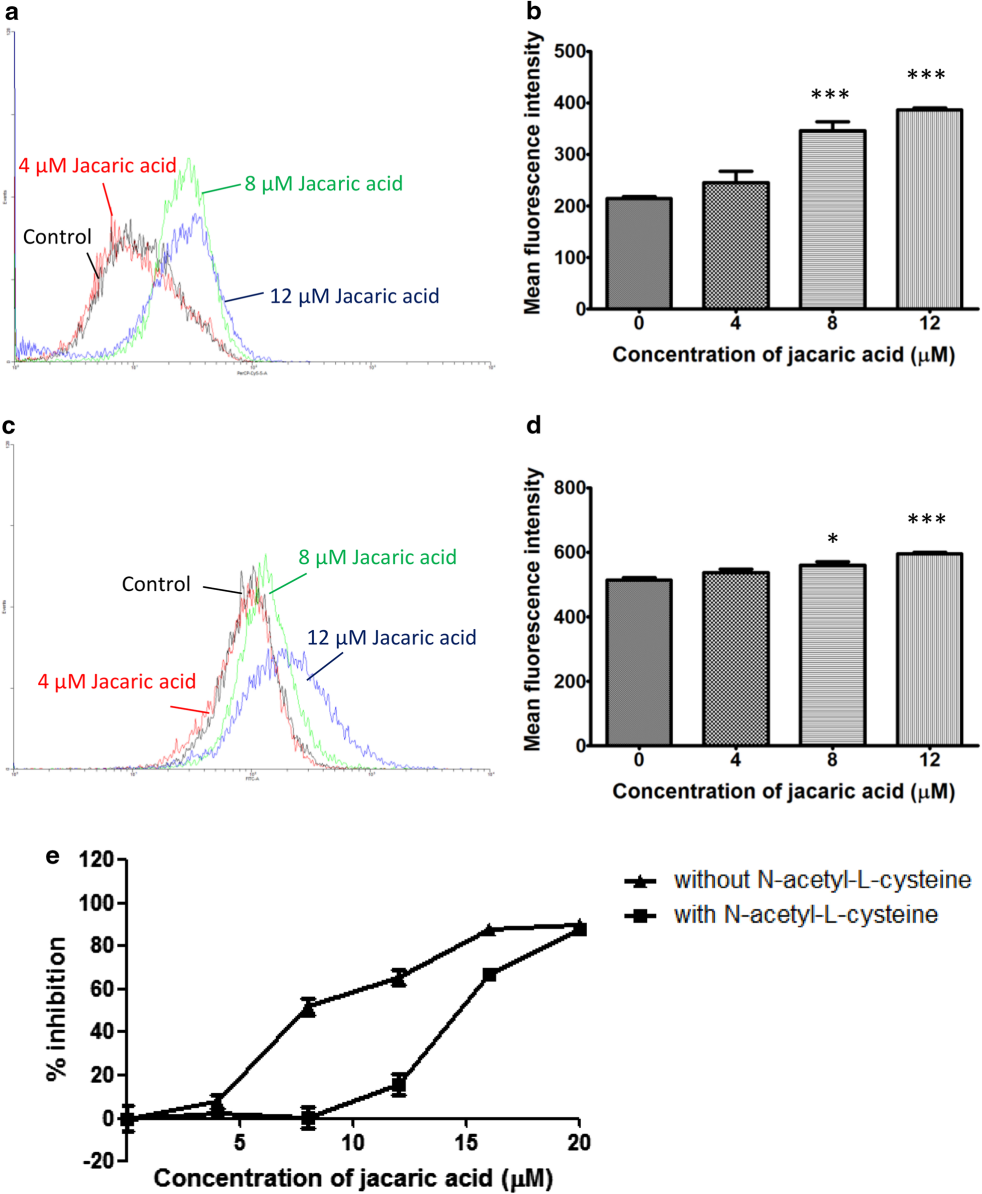
Effect of jacaric acid on the generation of intracellular ROS in PU5-1.8 cells. PU5-1.8 cells were incubated with different concentrations of jacaric acid at 37 °C for 48 h. **a**, **b** The intracellular levels of O_2_^−^ in the treated cells were measured by staining cells with DHE at 37 °C for 30 min and analyzed for red fluorescence (FL-3) by flow cytometry. Results were expressed as mean ± SE. ***p < 0.001. **c**, **d** The intracellular levels of H_2_O_2_ in the treated cells were measured by staining cells with H_2_DCFDA at 37 °C for 30 min and analyzed for green fluorescence (FL-1) by flow cytometry. Results were expressed as mean ± SE. *p < 0.05; ***p < 0.001. **e** PU5-1.8 cells were incubated with jacaric acid at 37 °C for 48 h, in the presence or absence of N-acetyl-l-cysteine. Cells treated with 0.01 % ethanol acted as the control. Cell growth was determined by the MTT reduction assay and the results were expressed as the mean % inhibition of cell proliferation ± SE

To determine the changes in the intracellular ROS levels in the jacaric-acid treated cells, the cells were stained by dihydroethidium (DHE) and 2′,7′-dichlorodihydrofluorescein diacetate (H_2_DCFDA) for the detection of superoxide anion (O_2_^−^) and hydrogen peroxide (H_2_O_2_) respectively. Flow cytometric analysis showed that the intracellular levels of O_2_^−^ (Fig. 4a, b) and H_2_O_2_ (Fig. 4c, d) were increased in a concentration-dependent manner. Interestingly, upon the addition of N-acetyl-l-cysteine, which is an antioxidant, the jacaric acid-induced anti-proliferative effect was suppressed (Fig. 4e). Taken together, the results suggested that ROS might play a role in mediating the anti-proliferative effect of jacaric acid on PU5-1.8 cells.

### Jacaric acid triggers cell cycle arrest at the G_0_/G_1_ phase and modulates the expression of cell cycle-regulatory proteins in PU5-1.8 cells

**Fig. 5:**
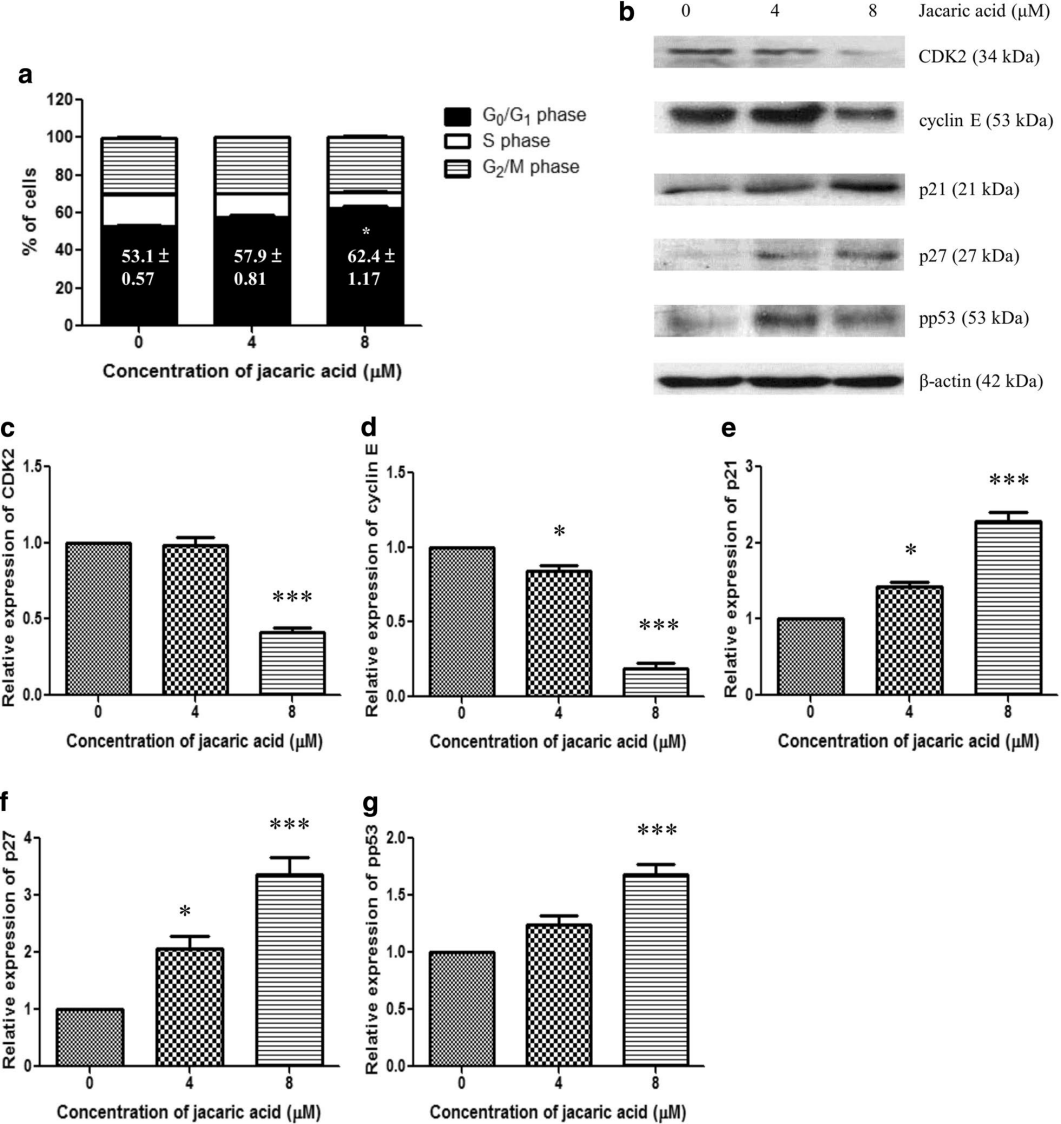
Modulation of cell cycle profile and cell cycle-regulatory proteins in jacaric acid-treated PU5-1.8 cells. **a** PU5-1.8 cells were incubated with different concentrations of jacaric acid at 37 °C for 72 h. Cells treated with ethanol acted as the control. The DNA content was analyzed by PI staining and flow cytometry. Cell cycle distribution of the samples was calculated by Modfit LT 3.0 programme. The results were expressed as mean ± SE. *p < 0.05. **b** PU5-1.8 cells were incubated with 4 μM jacaric acid (*Lane 2*) and 8 μM jacaric acid (*Lane 3*) at 37 °C for 72 h. Cells treated with ethanol (*Lane 1*) acted as the control. Protein expression levels of CDK2, cyclin E, p21, p27 and pp53 were assayed by Western blotting with β-actin protein as an internal control. **c**–**g** The relative protein expression levels of CDK2, cyclin E, p21, p27 and pp53 compared to β-actin were quantified. Results represent mean ± SE. *p < 0.05; ***p < 0.001

To determine the possible mechanisms of the anti-proliferative effect of jacaric acid on PU5-1.8 cells, cells were stained by propidium iodide (PI) after incubation with jacaric acid for 72 h, and the cell cycle profile was analyzed by flow cytometry. As shown in Fig. 5a, jacaric acid triggered cell cycle arrest at the G_0_/G_1_ phase, and accompanied by a decrease in the percentage of cells at the S phase. To further elucidate the underlying mechanisms, Western blotting was performed to examine the protein expression levels of cyclin-dependent kinase (CDK) 2, cyclin E, p21, p27 and pp53 (Fig. 5b), which are known to be involved in the transition of cell cycle from G_0_/G_1_ to S phase [18, 19]. Our results show that the protein expression levels of CDK2 and cyclin E decreased in jacaric acid-treated PU5-1.8 cells, whereas an elevation in the expression levels of the p21, p27 and pp53 proteins was observed (Fig. 5c–g). Collectively, the results indicate that jacaric acid treatment of PU5-1.8 cells could lead to the cell cycle arrest at the G_0_/G_1_ phase, and modulated the expression of certain cell cycle-regulatory proteins such as CDK2, cyclin E, p21, p27 and pp53.

### Jacaric acid inhibits the growth of PU5-1.8 cells by inducing apoptosis of the leukemia cells

**Fig. 6:**
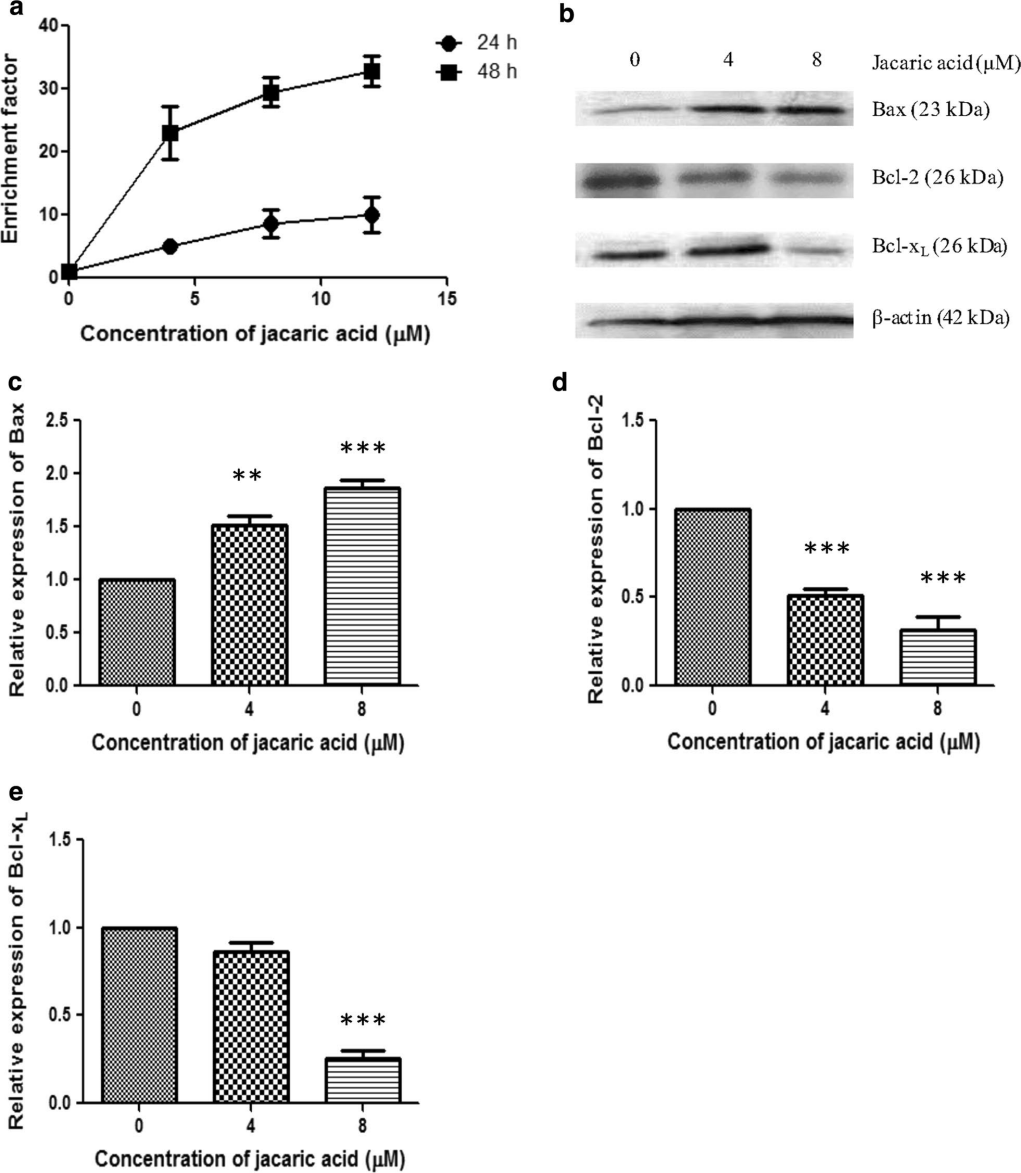
Induction of DNA fragmentation and modulation of apoptosis-regulatory proteins in jacaric acid-treated PU5-1.8 cells. **a** PU5-1.8 cells were incubated with different concentrations of jacaric acid at 37 °C for 24 or 48 h. Cells treated with ethanol acted as the control. DNA fragments were detected by using the Cell Death Detection ELISA^PLUS^ Kit. The extent of apoptosis in the samples was expressed as the enrichment factor. **b** PU5-1.8 cells were incubated with 4 μM jacaric acid (*Lane 2*) and 8 μM jacaric acid (*Lane 3*) at 37 °C for 72 h. Cells treated with ethanol (*Lane 1*) acted as the control. Protein expression levels of Bax, Bcl-2 and Bcl-x_L_ were assayed by Western blotting with β-actin protein as an internal control. **c**–**e** The relative protein expression levels of Bax, Bcl-2 and Bcl-x_L_ compared to β-actin were quantified. Results represent mean ± SE. **p < 0.01; ***p < 0.001

**Fig. 7:**
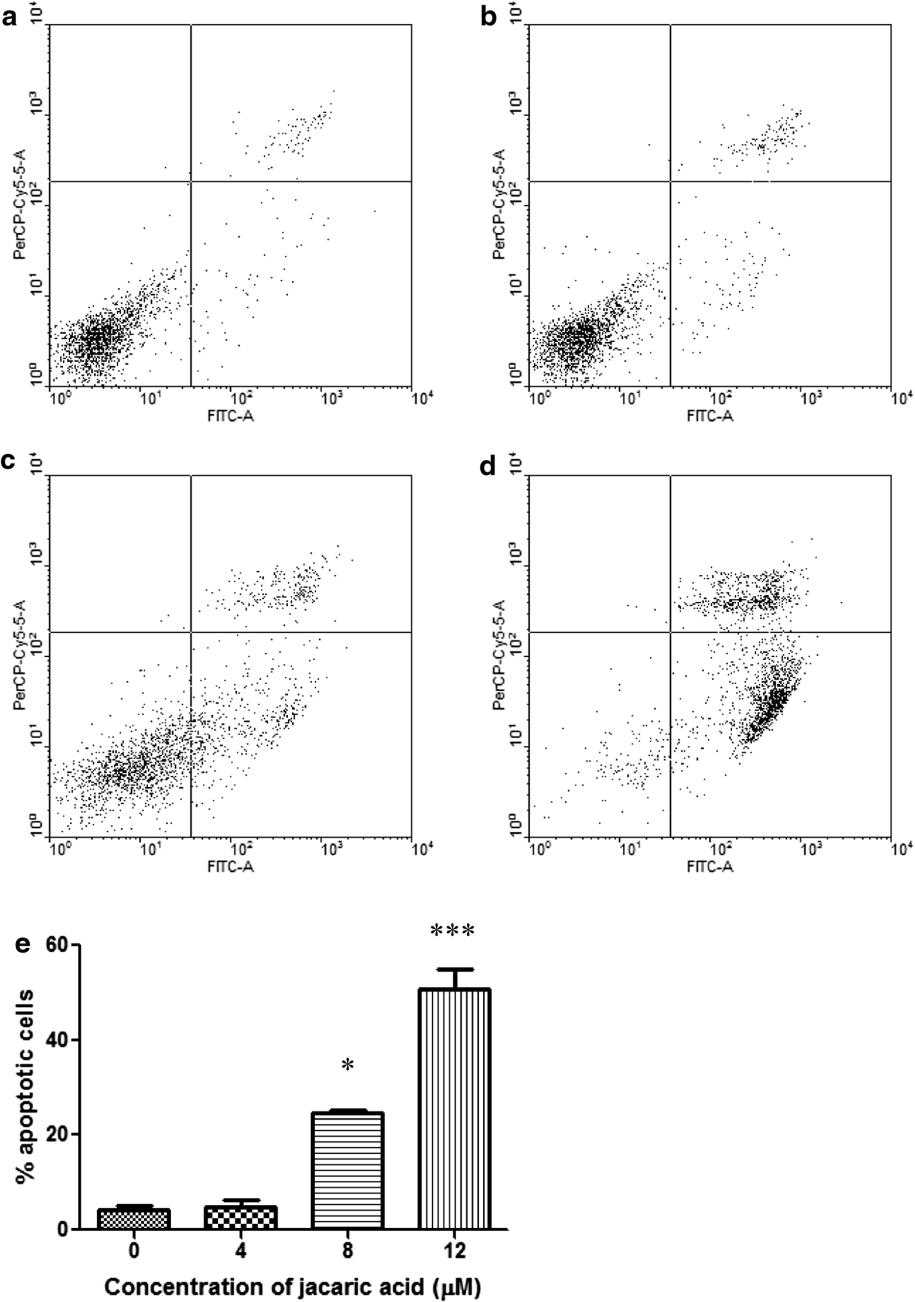
Jacaric acid induces phosphatidylserine externalization in PU5-1.8 cells. PU5-1.8 cells were incubated with either ethanol control (**a**), or 4 μM jacaric acid (**b**), 8 μM jacaric acid (**c**) and 12 μM jacaric acid (**d**) at 37 °C for 24 h. After incubation, the cells were stained with Annexin V-GFP fusion protein and PI. The fluorescence intensity was measured by the FACSCanto™ flow cytometer. **e** The results were quantified and expressed as mean ± SE. *p < 0.05; ***p < 0.001

**Fig. 8:**
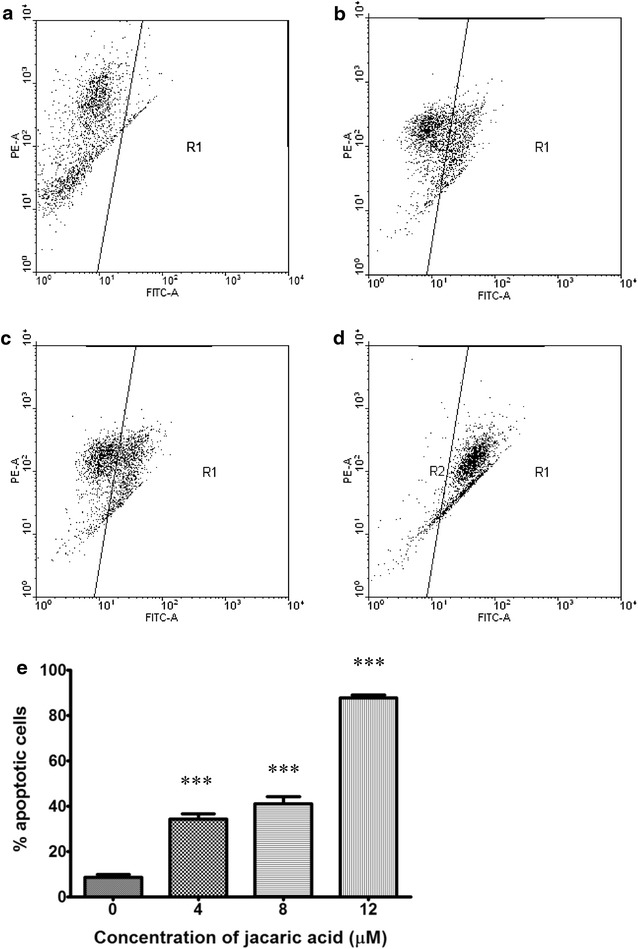
Jacaric acid induces mitochondrial membrane depolarization in PU5-1.8 cells. PU5-1.8 cells were incubated with either ethanol control (**a**), or 4 μM jacaric acid (**b**), 8 μM jacaric acid (**c**) and 12 μM jacaric acid (**d**) at 37 °C for 24 h. After incubation, the cells were stained with JC-1 dye. The fluorescence intensity was measured by the FACSCanto™ flow cytometer. **e** The results were quantified and expressed as mean ± SE. ***p < 0.001

Apart from triggering cell cycle arrest, another possible mechanism for the observed growth-inhibitory effect of jacaric acid is the induction of apoptosis. To examine whether jacaric acid would induce apoptosis in the PU5-1.8 cells, the Cell Death Detection ELISA^PLUS^ Kit was used according to the manufacturer’s instructions. It was found that jacaric acid could induce DNA fragmentation in the PU5-1.8 cells, as reflected by an increase in the enrichment factor in the jacaric acid-treated PU5-1.8 cells in a time- and concentration-dependent manner (Fig. 6a). To elucidate the molecular mechanisms underlying the induction of apoptosis, the effect of jacaric acid on altering the expression levels of different apoptosis-regulatory proteins were examined by Western blotting (Fig. 6b). It can be seen that the expression level of the pro-apoptotic Bax protein was increased, whereas the expression levels of the anti-apoptotic proteins Bcl-2 and Bcl-x_L_ were decreased (Fig. 6c–e). Apart from the Cell Death Detection ELISA, the Annexin V assay was used to detect the externalization of the phosphatidylserine (PS) which is a hallmark of early apoptosis [20]. In addition, JC-1 dye staining was used to measure the mitochondrial membrane potential which is known to decrease prior to PS externalization and DNA fragmentation [21, 22]. Our results show that jacaric acid could induce PS externalization in PU5-1.8 cells at 24 h of incubation in a concentration-dependent manner as demonstrated by the Annexin V assay (Fig. 7), and reduced the mitochondrial membrane potential of PU5-1.8 cells after staining with JC-1 dye (Fig. 8). These results, when taken together, suggest that jacaric acid could inhibit the growth of PU5-1.8 cells by inducing apoptosis.

## Discussion

The PU5-1.8 cell line used in the present study is a well-established murine macrophage-like leukemia [23–25], which has been commonly used as an in vitro model for the study of cell proliferative responses [26], apoptosis [27] and monocytic differentiation [28]. In the present study, we demonstrated that jacaric acid, one of the CLN isomers, could inhibit the growth of PU5-1.8 cells in a time- and concentration-dependent manner, as determined by the colorimetric MTT reduction assay and the fluorometric CyQUANT^®^ NF Cell Proliferation assay. Our results are similar to previous reports which demonstrated that CLN exerted potent anti-tumor effects on a wide range of human and murine cancer cell lines in vitro [29, 30]. Some previous reports suggested that the conjugated *all*-*trans* linolenic acid (β-calendic acid and β-eleostearic acid) had stronger growth-inhibitory effects on human epithelial colorectal adenocarcinoma Caco-2 cells [31] and human colon cancer HT-29 cells [32]. In the present study, it was found that among the six CLN isomers tested, jacaric acid but not β-calendic acid or β-eleostearic acid, exhibited the most potent anti-tumor effect on murine macrophage-like leukemia PU5-1.8 cells, and this is in line with the finding of Shinohara et al. [33] who also showed that jacaric acid exerted the most potent in vitro cytotoxic effect on the human adenocarcinoma DLD-1 cells. In addition, a previous report from our group also demonstrated that jacaric acid was more potent than other CLN isomers with regard to the anti-leukemic effect on human eosinophilic leukemia EoL-1 cells [34]. The discrepancy obtained from different studies might be the result of different cell models used.

Reports from other groups have shown that the oxidative stability of CLN is lower than that of their non-conjugated counterparts, as well as the CLA isomers [35, 36]. Tsuzuki et al. [37] reported that α-eleostearic acid, another CLN isomer, exhibited stronger anti-tumor effect than CLA on DLD-1 cells through lipid peroxidation, and the addition of antioxidant would suppress the oxidative stress and apoptosis. Similarly, Grossmann et al. [38] showed that the growth-inhibitory and apoptosis-inducing effects of *α*-eleostearic acid on human breast cancer cells are mediated through an oxidation-dependent mechanism. In the present study, the growth-inhibitory effect of jacaric acid on PU5-1.8 cells was reduced upon the addition of an antioxidant, N-acetyl-l-cysteine. On the other hand, flow cytometric analysis showed that jacaric acid could increase the intracellular levels of O_2_^−^ and H_2_O_2_ in a concentration-dependent manner. Therefore, it is conceivable that the oxidative stress induced by jacaric acid might be relieved in the presence of N-acetyl-l-cysteine. This provides an explanation for the ability of N-acetyl-l-cysteine to reduce the jacaric acid-induced growth inhibition in PU5-1.8 cells, and the results are in agreement with previous findings.

To investigate whether jacaric acid inhibited the growth of PU5-1.8 cells through triggering cell cycle arrest, the cells were stained by PI, and the cell cycle profile was analyzed by flow cytometry. Our results show that jacaric acid could trigger cell cycle arrest at the G_0_/G_1_ phase, and accompanied by a decrease in the percentage of cells at the S phase. Cell cycle progression was known to be regulated by different CDK and cyclins [39]. Some reports had shown that cell cycle arrest at G_0_/G_1_ phase was regulated by CDK2, CDK4 and cyclin E [39–41]. Other studies demonstrated that an increase in the protein expression levels of the p21, p27 and p53 proteins might cause cell cycle arrest at G_0_/G_1_ phase in human breast carcinoma and human lung cancer A549 cells [41, 42]. Our present study shows that the protein expression levels of CDK2 and cyclin E decreased in jacaric acid-treated PU5-1.8 cells, whereas there was an increase in the expression levels of the p21, p27 and pp53 proteins. Collectively, our results demonstrated that jacaric acid could lead to the cell cycle arrest at the G_0_/G_1_ phase, which was accompanied by an alteration of the cell cycle-regulatory proteins controlling the G_1_ phase mitotic check point.

Induction of apoptosis is another mechanism which might account for the observed anti-proliferative effect of jacaric acid on PU5-1.8 cells. Previous studies had demonstrated that *α*-eleostearic acid could induce DNA fragmentation, increase caspase activity and expression of caspase mRNA in the human colon cancer DLD-1 cells and these were shown to be associated with lipid peroxidation [37]. Some recent reports showed that β-eleostearic acid could induce apoptosis in human bladder cancer cells via a ROS-mediated pathway which might involve PPARγ activation [16] and jacaric acid could induce cell death through activation of intrinsic and extrinsic apoptotic pathways in human prostate cancer cells [17]. Using the Cell Death Detection ELISA^PLUS^ Kit, we found that there was an increase in DNA fragmentation in jacaric acid-treated PU5-1.8 cells, suggesting the occurrence of apoptosis in the leukemic cells. Western blotting indicated that the expression level of the pro-apoptotic protein Bax was increased, while the expression levels of the anti-apoptotic Bcl-2 and Bcl-x_L_ proteins were decreased, and these results are in line with the previous findings [43, 44]. In addition, an elevation in PS externalization and increased mitochondrial membrane depolarization in the jacaric acid-treated PU5-1.8 cells further supported our finding that jacaric acid could induce apoptosis in PU5-1.8 cells.

## Conclusions

The results, when taken together, suggest that jacaric acid can exhibit anti-proliferative activity on the murine macrophage-like leukemia PU5-1.8 cells by triggering cell cycle arrest at G_0_/G_1_ phase and by inducing leukemic cell apoptosis. A recent report showed that jacaric acid did not exhibit any significant toxicity in vivo [45], and our results also show that jacaric acid exhibited minimal cytotoxicity to the normal murine cells. Therefore, further elucidation of the anti-leukemic efficacy and action mechanisms of jacaric acid in vivo may provide better insights in the development of jacaric acid as a potential candidate for the treatment of some forms of myeloid leukemia with minimal toxicity and side effects.

## Methods

### Mice

Inbred female BALB/c (H-2^d^) mice aged 6–8 weeks old were obtained from the Laboratory Animal Services Centre of the Chinese University of Hong Kong and were kept in a specific pathogen-free condition. The animal experiments were conducted with the licence under Animals (Control of Experiments) Ordinance (Cap. 340) issued by the Department of Health of the Hong Kong Government, and according to the guidelines of the Animal Experimentation Ethics Committee, The Chinese University of Hong Kong, with the approval number 13/040/GRF.

### Chemicals and reagents

Conjugated linolenic acids used in the study, which include α-calendic acid (8*E*, 10*E*, 12*Z*-Octadecatrienoic acid), β-calendic acid (8*E*, 10*E*, 12*E*-Octadecatrienoic acid), jacaric acid (8*Z*, 10*E*, 12*Z*-Octadecatrienoic acid), α-eleostearic acid (9*Z*, 11*E*, 13*E*-Octadecatrienoic acid), β-eleostearic acid (9*E*, 11*E*, 13*E*-Octadecatrienoic acid) and punicic acid (9*Z*, 11*E*, 13*Z*-Octadecatrienoic acid) (Fig. 1a–f), all with an estimated purity >97 %, were purchased from Larodan Fine Chemicals AB, Sweden. The stock solution (0.2 M) was prepared by dissolving the powder in cell culture-tested ethanol (Sigma-Aldrich Co., USA). All other chemicals were purchased from Sigma-Aldrich unless otherwise stated.

### Culture of murine leukemia cell lines and normal murine cells

PU5-1.8 cell line is a macrophage-like tumor cell line which was originally derived from a spontaneous tumor in BALB/c mice which had been adapted to grow in culture [24]. J774 A.1 is a macrophage-like tumor cell line which was derived from a female BALB/c mouse [46]. P388D1 is a macrophage-like tumor cell line which was derived from the methylcholanthrene-induced tumor in DBA/2 mouse [46]. These macrophage-like leukemia cell lines were purchased from the American Type Culture Collection (ATCC) in USA. The murine leukemia cells were maintained in RPMI-1640 medium (GIBCO, USA) supplemented with 10 % fetal bovine serum (FBS; GIBCO, USA) and 1 % antibiotics (100 units/ml penicillin G, 100 µg/ml streptomycin sulfate and 0.25 μg/ml amphotericin B in 0.85 % saline) in a humidified incubator containing 5 % CO_2_ at 37 °C.

Normal murine cells, including bone marrow cells, peritoneal macrophages, splenocytes and thymocytes were freshly isolated from inbred female BALB/c mice aged 6–8 weeks old. In our experiments, the normal murine cells were seeded into the wells of a 96-well microtiter plate (2.5 × 10^6^/ml) in RPMI medium supplemented with 10 % fetal bovine serum and 1 % antibiotics and they were incubated at 37 °C with different concentrations of jacaric acid for 48 h. Cells treated with 0.1 % ethanol acted as the control. The cell viability was measured by the standard colorimetric (3-(4,5-dimethylthiazol-2-yl)-2,5-diphenyltetrazolium bromide) (MTT) reduction assay [34].

### In vitro assays for the anti-proliferative activity

The colorimetric MTT reduction assay was used to measure cell proliferation [34]. Briefly, the murine macrophage-like leukemia cells (2.5 × 10^4^/ml) were seeded into the wells of a 96-well microtiter plate and incubated with different concentrations of jacaric acid at 37 °C for various periods of time. Cells treated with 0.01 % ethanol acted as the control. The anti-proliferative response of jacaric acid on the leukemia cells was measured by the MTT assay, and the absorbance at 540 nm (OD_540_) was recorded by a BENCHMARK microplate reader (Bio-Rad Laboratories, USA). The results were confirmed using the CyQUANT^®^ NF Cell Proliferation Assay Kit (Molecular Probes; Invitrogen Corp., USA) as previously described [34], and the fluorescence intensity was recorded by a fluorescence plate reader (Tecan Polarion, USA).

### Analysis of ROS levels

PU5-1.8 cells (2.5 × 10^4^/ml) were treated with different concentrations of jacaric acid at 37 °C for 48 h in 75 cm^2^ tissue culture flask. The cell pellet was washed once with PBS and then resuspended in 0.5 ml PBS containing 10 µM DHE or H_2_DCFDA and incubated at 37 °C in dark for 30 min with gentle shaking. Afterwards, the cells were harvested and analyzed for red fluorescence (FL-3) with an excitation wavelength of 488 nm and an emission wavelength of 670 nm (for detection of DHE) or for green fluorescence (FL-1) with an excitation wavelength of 488 nm and an emission wavelength of 530 nm (for detection of H_2_DCFDA) by flow cytometry (FACSCanto™ flow cytometer, BD BioSciences, USA).

### Analysis of cell cycle profile

PU5-1.8 cells (2.5 × 10^4^/ml) were synchronized by incubation of the cells with plain RPMI medium supplemented with 0.5 % heat-inactivated FBS in 75 cm^2^ tissue culture flask at 37 °C overnight. Cells readjusted to a density of 2.5 × 10^4^ cells/ml were then incubated with different concentrations of jacaric acid at 37 °C for 72 h. Afterwards, the cells were fixed by 1 ml 70 % ethanol at 4 °C for 30 min, and then stained by PI (40 μg/ml) (Sigma-Aldrich Co., USA) at 37 °C in dark for 30 min with gentle shaking. The cell cycle profile of the stained cells was analyzed by the FACSCanto flow cytometer using the software ModFit LT V3.0 (Verity Software House).

### Measurement of DNA fragmentation by Cell Death Detection ELISA^PLUS^ Kit

The measurement was performed according to the manufacturer’s instructions in the Cell Death Detection ELISA^PLUS^ Kit (Roche Applied Science, USA). Briefly, PU5-1.8 cells (2.5 × 10^4^/ml) were seeded into the wells of a flat-bottomed 96-well plate and incubated with different concentrations of jacaric acid at 37 °C for various periods of time. The supernatant was transferred to the well of the streptavidin-coated 96-well microtiter plate and the absorbance at 405 nm (OD_405_) was recorded by the BENCHMARK microplate reader. The degree of apoptosis was expressed as enrichment factor, which was calculated as follows:

$${\text{Enrichment factor}}\,{ = }\,\frac{{{\text{Absorbance of the jacaric acid - treated}}\,{\text{cells}}}}{{{\text{Absorbance of the control}}\,{\text{cells without jacaric acid treatment}}}}.$$

### Analysis of Annexin V-GFP/PI dual staining profile

PU5-1.8 cells (2.5 × 10^4^/ml) were incubated with different concentrations of jacaric acid at 37 °C for 24 h in 75 cm^2^ tissue culture flask. The cells were then resuspended in Annexin V binding buffer (BD Biosciences, USA) supplemented with Annexin V-Green Fluorescence Protein (GFP) fusion protein (1 µg/ml) and PI (4 µg/ml). The samples were incubated at room temperature for 30 min and were analyzed for red fluorescence (for PI staining) with an excitation wavelength of 488 nm and an emission wavelength of 670 nm, versus green fluorescence (for GFP staining) with an excitation wavelength of 488 nm and an emission wavelength of 530 nm using the FACSCanto™ flow cytometer. The percentages of cells at the four quadrants were calculated by the WinMDI (Version 2.9) software.

### Determination of mitochondrial membrane potential by JC-1 staining

PU5-1.8 cells (2.5 × 10^4^/ml) were incubated with different concentrations of jacaric acid at 37 °C for 24 h in 75 cm^2^ tissue culture flask. Afterwards, the cells were resuspended in PBS supplemented with 10 µg/ml JC-1 dye (Molecular Probes, Invitrogen Corporation, USA). The samples were incubated at 37 °C for 30 min and then analyzed for red fluorescence (FL-2) with an excitation wavelength of 488 nm and an emission wavelength of 585 nm versus green fluorescence (FL-1) with an excitation wavelength of 488 nm and an emission wavelength of 530 nm using the FACSCanto™ flow cytometer. The percentages of cells with membrane depolarization were calculated by the WinMDI (Version 2.9) software.

### Western blot analysis

Protein expression was determined by Western blotting technique using a panel of specific antibodies. Briefly, PU5-1.8 cells (2.5 × 10^4^/ml) were incubated with different concentrations of jacaric acid at 37 °C for 72 h in 175 cm^2^ tissue culture flasks. Cell pellets were collected and total proteins were extracted by the cell lysis buffer. Protein concentrations were measured by Bradford reagent (Sigma-Aldrich Co., USA) and the protein samples were resolved on 12 % polyacrylamide gels and transferred to PVDF membranes. Membranes were first incubated with the following primary antibodies: mouse anti-Bax, anti-Bcl-2, anti-Bcl-x_L_, anti-p21, anti-p27 and anti-CDK2 antibodies (Santa Cruz Biotechnology, USA), rabbit anti-cyclin E antibody (Santa Cruz Biotechnology, USA), rabbit anti-pp53 antibody (Cell Signaling Technology, USA) and mouse anti-β-actin antibody (Sigma-Aldrich Co., USA), followed by incubation with horseradish peroxidase (HRP)-conjugated secondary antibodies (GE Healthcare Limited, UK) and finally developed with the enhanced chemiluminescence (ECL) reagent (Santa Cruz Biotechnology, USA).

### Statistical analysis

Each experiment was repeated three times and only the results of the most representative experiments are shown. The data are expressed as the arithmetic mean ± standard error (SE). One-way analysis of variance (ANOVA) with post hoc Tukey’s Multiple Comparison Test was used for statistical analysis and the differences were considered as statistically significant at *p* < 0.05.

## Abbreviations

CDK: cyclin-dependent kinase
CFA: conjugated fatty acids
CLA: conjugated linoleic acids
CLN: conjugated linolenic acids
DHE: dihydroethidium
ECL: enhanced chemiluminescence
FBS: fetal bovine serum
H_2_DCFDA: dichlorodihydrofluorescein diacetate
HRP: horseradish peroxidase
HSC: hematopoietic stem cells
PI: propidium iodide
PS: phosphatidylserine
PVDF: polyvinylidene difluoride
